# Acute Urinary Retention and Severe Hyponatremia in a Patient With a Large Intramural Uterine Fibroid

**DOI:** 10.7759/cureus.68587

**Published:** 2024-09-03

**Authors:** Hoore Jannat, Syed Sadam Hussain, Hamad Ahmad

**Affiliations:** 1 Internal Medicine, Khyber Medical University, Peshawar, PAK; 2 Internal Medicine, Westchester Medical Center, Valhalla, USA; 3 Internal Medicine/Malignant Hematology, Westchester Medical Center, Valhalla, USA

**Keywords:** urinary bladder decompression, syndrome of inappropriate secretion of antidiuretic hormone (siadh), uterine fibroid, severe hyponatremia, acute urinary retention (aur)

## Abstract

Acute urinary retention (AUR) is defined as the inability to pass urine voluntarily. It is more common in males, with a male-to-female incidence ratio of 13:1. In males, benign prostatic hyperplasia is the most common cause of AUR, especially in men aged above 60, whereas in females, pelvic anatomy distortion secondary to pelvic organ prolapse and pelvic masses causes most cases of AUR. Prompt diagnosis and management are the keys to avoiding complications secondary to AUR, such as pain and acute kidney injury. Less commonly, it can cause acute hyponatremia, as was seen in our patient. Hyponatremia is generally asymptomatic, but if acute and/or severe, it can cause mental status changes, seizures, and coma. Such patients need closer monitoring of their mental status and sodium level to avoid overcorrection. Here we present a unique case report of a patient with asymptomatic large uterine fibroid presented with abdominal distention who was found to have acute urinary retention with associated asymptomatic severe hyponatremia, managed conservatively.

## Introduction

Acute urinary retention (AUR) is defined as the inability to pass urine voluntarily. It is more common in males, with a male-to-female incidence ratio of 13:1 [1]. Benign prostatic hyperplasia is the single most common cause in males [2], whereas in females, AUR generally results from anatomic distortion secondary to pelvic organ prolapse, pelvic masses, and less commonly, urethral diverticulum [3]. If not diagnosed and treated promptly, AUR can result in acute kidney injury, hematuria, urinary tract infection, loss of bladder contractility, and, less commonly, electrolyte disturbances such as hyponatremia [4]. Hyponatremia, defined as a serum sodium level of less than 135 meq/L, is the most common electrolyte disorder and is associated with increased hospital stay and mortality [5]. Hyponatremia is considered acute if it develops over a period of less than 48 hours and is considered severe if the serum concentration of sodium falls below 120. Symptoms vary from being asymptomatic to mental status changes, seizures, coma, and respiratory arrest [6]. Patients with more acute-onset and severe hyponatremia tend to be high-risk and symptomatic and need aggressive management, including sodium correction with hypertonic saline and addressing the underlying etiology [7]. If asymptomatic, a conservative approach with correction of the underlying etiology and closer monitoring is generally sufficient. Hyponatremia in the setting of acute urinary retention is thought to be due to the release of vasopressin triggered by bladder distension itself or by pain due to bladder distension [8]. 

## Case presentation

**Figure 1:**
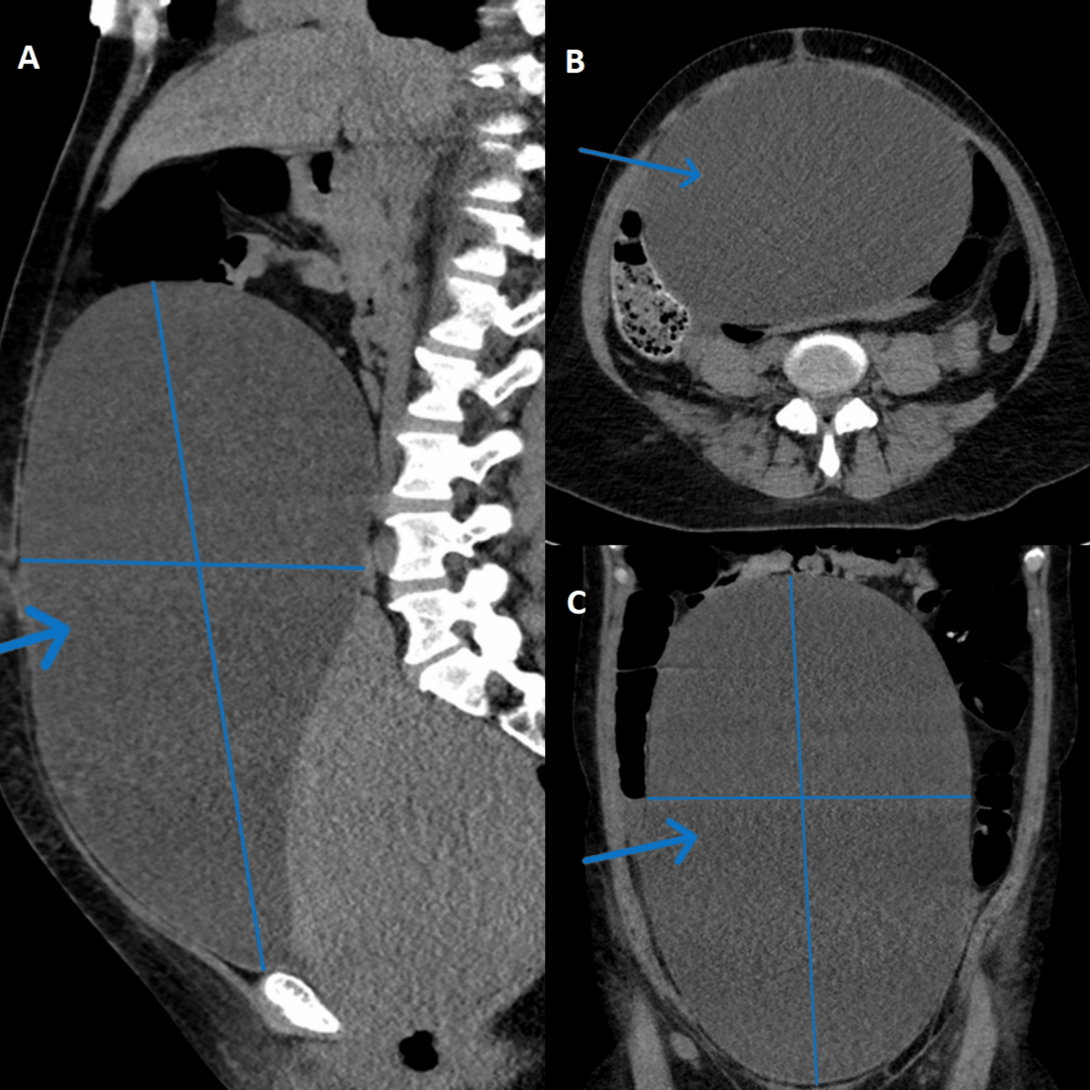
Blue arrows in pictures A, B, and C point to an enlarged urinary bladder in sagittal, cross-sectional, and coronal views, respectively, in the non-contrast computed tomography (CT) scan of the abdomen and pelvis.

We present a 50-year-old female with no significant past medical history who presented to the emergency department for the evaluation of acute abdominal distention, progressive with associated abdominal discomfort for almost a week; otherwise, no associated nausea, diarrhea, dysuria, hematuria, melena, hematochezia, or fever. No new medications or any history of prior medication use on a daily basis. On examination, the abdomen was distended and mildly tender to palpation, though there was no guarding or rigidity. Bowel sounds were normal. The rest of the systemic examination was unremarkable. Blood pressure 128/70 mmHg, pulse rate 80 beats per minute, respiratory rate 11 breaths per minute, and oxygen saturation 95% on room air. Blood work was significant for a white blood cell (WBC) count of 14,600 cells/mm³ (4000-11000 cells/mm^3^), sodium level of 118 mEq/L (135-145 mEq/L), chloride level of 84 mEq/L (96-106 mEq/L), blood urea nitrogen (BUN) of 25 mg/dL (7-20 mg/dl), creatinine (Cr) of 2.16 mg/dL (0.6-1.2 mg/dL) and an anion gap of 14 mEq/L (4-12 mEq/L) with a normal lactate level. Urinalysis showed yellow-colored, clear urine with a pH 5.5, a specific gravity of 1.010, ketones: none, nitrites: negative, leukocyte esterase: negative, bilirubin: negative, blood: three RBCs, protein: negative, WBCs: two WBCs/hpf, casts: negative, and bacteria: none. Blood culture and urine culture did not grow any organisms. A non-contrast computed tomography (CT) scan of the abdomen and pelvis showed a large cystic structure in the lower abdomen and pelvis, concerning a pelvic mass and/or distended urinary bladder (Figure 1).

**Figure 2:**
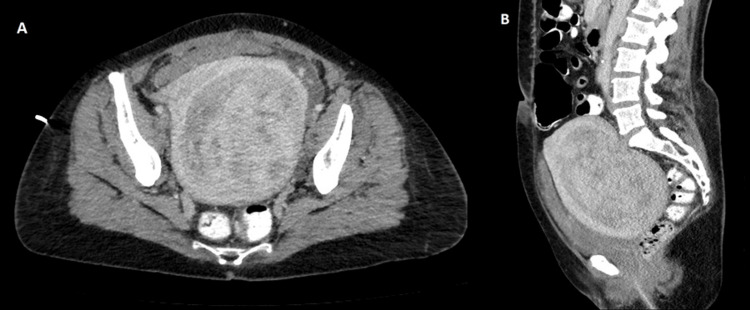
Computed tomography (CT) scan with intravascular contrast confirmed a large heterogeneously enhancing uterine mass, which may be intramural or endometrial in origin, seen in cross-sectional and sagittal views in pictures A and B respectively.

Renal bladder ultrasound showed a severely distended urinary bladder with mild left-sided hydronephrosis. In the emergency room, patient received 1 liter of normal saline fluid. Four liters of urine were drained immediately upon urinary catheter placement. Patient felt much better afterward and discharged herself against medical advice. Ten hours later, patient presented again to the emergency room with a similar complaint. Examination was similar to before, though abdominal distention and tenderness had resolved. Repeat blood work showed normal WBC count of 10,000 cells/mm³, normal BUN of 22 mg/dL, normal creatinine of 1.0 mg/dL, and normal serum sodium of 135 mEq/L. Given the abrupt correction of hyponatremia, the patient was started on a dextrose water infusion to slow down the correction rate to avoid complications. Electrolytes were closely monitored every four hours, and the sodium level remained stable in the mid-130s mEq/L. Patient remained clinically stable, awake, and alert at baseline. Further workup for the etiology of urinary retention and possible pelvic mass was carried out. A repeat CT with contrast confirmed a large heterogeneously enhancing uterine mass, which may be intramural or endometrial in origin (Figure 2).

**Figure 3:**
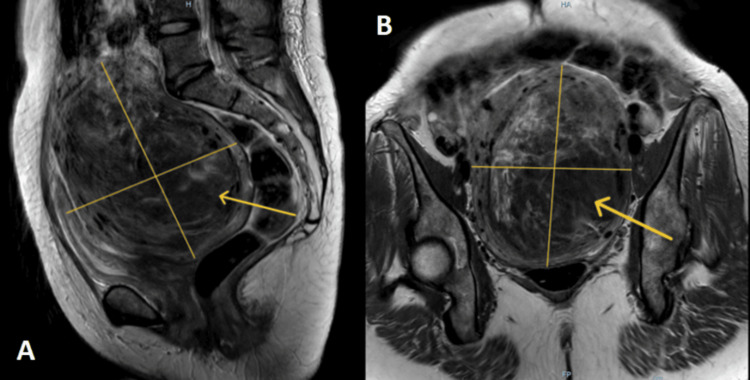
Magnetic resonance imaging (MRI) of the abdomen pelvis shows a large 13.1 cm intramural uterine fibroid Grade 4 (yellow arrows).

An MRI pelvis was done for further characterization of the mass, which showed a large 13.1 cm intramural uterine fibroid Grade 4 (Figure 3). A biopsy of the lesion confirmed fibroids with no evidence for malignancy. Patient was discharged and had a hysterectomy as an outpatient.

## Discussion

Acute urinary retention is a cause of significant morbidity in the elderly population, predominantly affecting males. In contrast, AUR incidence in females is significantly low, estimated at three cases per 100,000 per year [2]. The etiology of AUR is poorly understood; possible mechanisms involved are outflow obstruction, neurologic impairment, or an inefficient detrusor muscle [9]. Our patient being a female, age less than 60, with no prior history of urinary retention or any known risk factors mentioned above made her situation tricky. The most common presenting symptoms are lower abdominal discomfort, urinary tract infection, overflow incontinence, and declining renal function with decreased urine output [10]. Our patient's main complaint was abdominal distention, which is not a very typical presentation. It is reported that patients may not have significant pain if there is a component of chronic urinary retention [11].

AUR is a urologic emergency that requires immediate treatment by the insertion of a urinary catheter that allows the bladder to empty. Upon workup and suspicion for urinary retention, our patient had an emergent Foley catheter placed after ruling out any contraindications to bladder decompression. It is recommended that if the urine volume removed is greater than 400 ml and/or patient has an acute kidney injury, the catheter should be left in place [12]; hence, the catheter was kept in place. Hematuria, transient hypotension, and post-obstructive diuresis may develop after bladder decompression, but our patient did not have any of these complications [12]. Since the underlying etiology on further workup was found to be secondary to pelvis mass, it was later confirmed to be a large uterine fibroid by MRI pelvis and biopsy. Though our patient was otherwise asymptomatic from fibroids, she underwent a laparoscopic hysterectomy as she remains at high risk for recurrent urinary retention, and it is recommended that the underlying risk factor be addressed to reduce the likelihood of urinary retention in the future [13].

Since our patient developed severe hyponatremia as a result of AUR, we closely observed the electrolytes and patient's clinical status in a hospitalized setting. It is recommended that patients who are symptomatic and need aggressive management be transferred to the intensive care unit [7]. Hyponatremia in the setting of acute urinary retention is believed to be secondary to excess antidiuretic hormone (ADH) release due to severe bladder wall stretch and pain [8]. Patient is euvolemic, and addressing the underlying urinary retention and rectifying it results in hyponatremia resolution. Our patient received one liter of normal saline in the emergency room, followed by bladder decompression; a follow-up basal metabolic panel 12 hours later showed a sodium level of 135; hence, a dextrose water infusion was started to slow down the correction rate further as it had already been overcorrected. Had our patient not left against medical advice and had more frequent monitoring, overcorrection could have been avoided. During the treatment phase, patients should be monitored for signs of increased intracranial pressure (ICP), especially those with a high risk of brain herniation if overcorrection happens [7]. Our patient remained clinically stable, coherent, alert, and oriented, with no signs of increased ICP.

## Conclusions

Our case highlights the critical need for recognizing AUR in females, who present atypically with symptoms like abdominal distention, and underscores the necessity for immediate bladder decompression to prevent complications such as acute kidney injury and severe hyponatremia. Key recommendations include prompt identification and treatment of underlying causes to prevent recurrence, vigilant monitoring of electrolyte levels to avoid overcorrection, and ensuring continuous patient observation, particularly for those at risk of rapid electrolyte changes. To mitigate complications, it is imperative that patients avoid self-discharge against medical advice. Future research should aim to identify early markers and risk factors for AUR in females, develop optimal strategies for managing associated hyponatremia, and evaluate long-term outcomes and treatment efficacy for preventing recurrence in patients with AUR due to pelvic masses.

## References

[1] Ramsey S, Palmer M (2006). The management of female urinary retention. Int Urol Nephrol.

[2] Jacobsen SJ, Jacobson DJ, Girman CJ (1997). Natural history of prostatism: risk factors for acute urinary retention. J Urol.

[3] Klarskov P, Andersen JT, Asmussen CF (1987). Acute urinary retention in women: a prospective study of 18 consecutive cases. Scand J Urol Nephrol.

[4] Xie N, Hu Z, Ye Z, Xu Q, Chen J, Lin Y (2021). A systematic review comparing early with late removal of indwelling urinary catheters after pelvic organ prolapse surgery. Int Urogynecol J.

[5] Adrogué HJ, Tucker BM, Madias NE (2022). Diagnosis and management of hyponatremia: a review. JAMA.

[6] Sterns RH (1987). Severe symptomatic hyponatremia: treatment and outcome. A study of 64 cases. Ann Intern Med.

[7] Sterns RH (2018). Treatment of severe hyponatremia. Clin J Am Soc Nephrol.

[8] Parikh J, Dhareshwar S, Nayak-Rao S, Ramaiah I (2017). Hyponatremia secondary to acute urinary retention. Saudi J Kidney Dis Transpl.

[9] Choong S, Emberton M (2000). Acute urinary retention. BJU Int.

[10] Thomas K, Chow K, Kirby RS (2004). Acute urinary retention: a review of the aetiology and management. Prostate Cancer Prostatic Dis.

[11] Blackburn T, Dunn M (1990). Cystocerebral syndrome. Acute urinary retention presenting as confusion in elderly patients. Arch Intern Med.

[12] Nyman MA, Schwenk NM, Silverstein MD (1997). Management of urinary retention: rapid versus gradual decompression and risk of complications. Mayo Clin Proc.

[13] Fitzgerald MP, Kulkarni N, Fenner D (2000). Postoperative resolution of urinary retention in patients with advanced pelvic organ prolapse. Am J Obstet Gynecol.

